# The Effect of Heparin and Its Preparations on Disseminated Intravascular Coagulation Mortality and Hospitalization: A Systematic Review

**DOI:** 10.1155/2022/2226761

**Published:** 2022-07-09

**Authors:** Navid Omidkhoda, Farshad Abedi, Vahid Ghavami, Hossein Rahimi, Sara Samadi, Omid Arasteh, Amir Hooshang Mohammadpour

**Affiliations:** ^1^Department of Clinical Pharmacy, School of Pharmacy, Mashhad University of Medical Sciences, Mashhad, Iran; ^2^Department of Biostatistics, School of Health, Mashhad University of Medical Sciences, Mashhad, Iran; ^3^Department of Internal Medicine, Ghaem Hospital, Mashhad University of Medical Sciences, Mashhad, Iran; ^4^Pharmaceutical Research Center, Pharmaceutical Technology Institute, Mashhad University of Medical Sciences, Mashhad, Iran

## Abstract

**Methods:**

The databases of PubMed, Scopus, Embase, and Web of Science were searched systematically up to November 2021. The quality of RCTs was assessed by Cochrane Collaboration's tool and the risk of bias was assessed for cohort studies through NOS score.

**Results:**

Out of 3288 articles, eight studies were eligible to be included in this study. Our review retrieved six RCTs and two retrospective cohort studies consisting of 950 participants diagnosed by DIC. A significant effect of heparin on DIC mortality was identified in four studies. Furthermore, heparin was used as a control group in three studies.

**Conclusions:**

We concluded that administration of heparin and its preparations in DIC patients could reduce the mortality rate and duration of hospitalization, especially in the earlier stages of DIC.

## 1. Introduction

**Figure 1:**
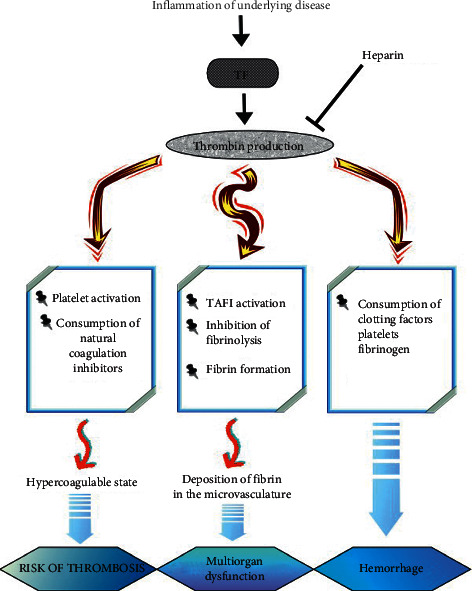
Pathogenetic pathways in DIC. Activation of coagulation is driven by TF overexpression leading to explosive and disseminated thrombin generation, which results in the consumption of natural coagulation inhibitors (mainly AT and PC) and in a hypercoagulable state. Thrombin, among other inducers, enhances platelet activation. Activated platelets amplify hypercoagulable state. Inhibition of fibrinolysis, through TAFI activation, increases fibrin formation and deposition in the microvasculature. This mechanism—among others—is implicated in the pathogenesis of organ dysfunction and multiorgan failure. Sustained thrombin generation has, as a consequence, the consumption of clotting factors, platelets, and fibrinogen. Severe clotting factor and fibrinogen deficiency together with severe thrombocytopenia are in the origin of the hemorrhagic syndrome in DIC. AT: antithrombin; DIC: disseminated intravascular coagulation; PC: protein C; and TF: tissue factor.

Disseminated intravascular coagulation (DIC) is considered as a crucial medical condition that is expressed by systemic activation of the homeostatic system and result in elevation of thrombin deposition and microvascular thrombi [1–3]. Furthermore, platelets consumption, degeneration of intravascular fibrin, and imbalance between the antifibrinolytic and fibrinolytic systems can also cause severe bleeding [4]. Indeed, various pathological situations such as sepsis, trauma, cancer, surgery, and hepatic disease may induce DIC (Table 1) [5, 6]. Several mechanisms have been proposed to trigger the DIC including tissue factor (TF) overexpression, disproportionate thrombin production, flaws in the function of natural anticoagulants, excessive fibrin/fibrinogen degradation, and accompanying inflammatory process activation (Figure 1) [7, 8]. Nevertheless, since rapid recovery from the underlying disease cannot be seen in all patients, it is reasonable to manage the state of extreme hypercoagulability with anticoagulants to reduce intravascular coagulation activation [9]. The most common and available treatment among the anticoagulant medications is heparin and its preparations to manage the hypercoagulability situation in DIC. Due to the heparin's major activity in inhibition of thrombin, a key component in the DIC pathogenesis (Figure 1), it makes sense to prioritize heparin for pharmacotherapy [10]. Additionally, protamine as an antidote of heparin with suitable effect is available in the pharmaceutical market [11]. However, there are some reports that indicated administration of heparin and its preparations could be worsening the hemorrhage and raise the mortality rate, which makes the safety and practicability of these treatments controversial [12]. Wen et al. have investigated the effect of heparin and low-molecular-weight heparin (LMWH) separately against control group on traumatic disseminated intravascular coagulation. They reported a significant difference in mortality rate in control group in comparison with treatment groups [13]. Also, in a study by Ning et al., the impacts of heparin and LMWH on patients infected with coronavirus and high risk of DIC were evaluated. As they have reported, a significant reduction was observed in a 28-day mortality rate in patients treated by heparin and LMWH in comparison with nontreating heparin group [14]. Although previous evidences have suggested the positive role of heparin in DIC, another study has shown 83 percent mortality rate in heparin group, in comparison with 86 percent mortality rate in non-heparin-treated patients, which indicated no significant difference between the two groups [15].

Considering the important role of heparin and its preparations in DIC mortality and duration of hospitalization, it is very beneficial to evaluate the results of randomized clinical trials and cohort studies to achieve an evidence-based conclusion in this regard.

## 2. Methods

This systematic review was registered with PROSPERO (CRD42021260261) and performed in accordance with the Preferred Reporting Items for Systematic Reviews and Meta-Analyses (PRISMA) guidelines [16].

### 2.1. Eligibility Criteria

In this systematic review, the retrospective cohort studies and interventional studies were evaluated at the beginning against the eligibility criteria. The type of the study was limited to human studies including both randomized controlled clinical trials (RCTs) and cohort studies. The inclusion criteria were based on PICOS, as follows: P (Participants): patients diagnosed as having DIC regardless of their race, gender, and age; I (Intervention): received heparin or its preparations; and O (Outcome): the main outcome was the mortality rate and duration of hospitalization.

Studies were excluded for the following reasons: duplicated articles, review articles, personal opinions, book chapters, conference abstracts, and animal studies.

### 2.2. Search Strategy and Data Extraction

The scientific databases of PubMed, Scopus, Embase, and Web of Science were searched systematically up to November 2021 to identify relevant clinical trials about the effect of heparin and its preparations on DIC mortality and hospitalization. We applied a mixture of the Medical Subject Headings (MESH) and non-MESH words to identify research because of increasing sensitivity and specificity. The following keywords were chosen: “Disseminated intravascular coagulation” OR “consumption coagulopathy” AND “Heparin” OR “Unfractionated Heparin” OR “Low-Molecular-Weight Heparin” AND “mortality rate^*∗*^” OR “Hospitalization.” The complete search strategy is in the supplementary file. During the search for the listed databases, no language or time restriction was considered. Furthermore, we hand-searched and scrutinized the reference lists of all included original literature and checked them to find any potentially qualifying publications using the search terms. Finally, two reviewers investigated the search results and the titles and abstracts of the retrieved articles, independently. Then, irrelevant studies were excluded and the full text of all potentially relevant studies was sought and thoroughly read by the two authors. Furthermore, a 3rd author participated in resolving any disagreements regarding the data extraction between two authors.

The following data were extracted by reviewers: authors, year of publication, type of article and study design, country, duration of the study, underlying disease (cause of DIC), sample size in case and control groups, and age of the participants, intervention and dose of intervention, and the main outcomes.

### 2.3. Quality and Risk of Bias Assessment

The risk of bias was assessed using the Newcastle-Ottawa Scale (NOS) for assessing cohort studies. The NOS includes 3 parts: selection domain with 4 questions, comparability domain with 1 question, and outcome domain with 3 questions. The NOS assigns a maximum of 4 stars for selection, 2 stars for comparability, and 3 stars for exposure/outcome. Therefore, the highest-quality study gets 9 stars. The assessing risk of bias in randomized trials conducted by Cochrane Collaboration's tool for each identified study. This tool assesses the likelihood of bias in randomized trials, including the adequate generation of allocation sequence, acceptable concealment of allocation, acceptable blinding of participants, personnel and outcome assessors, and analyzing the risk of bias in reporting outcome data. Two authors independently assessed the risk of bias for each eligible study. A 3rd author took part in resolving any disagreements regarding the risk of bias assessment between two authors [17].

## 3. Results

### 3.1. General Characteristics of the Studies

**Figure 2:**
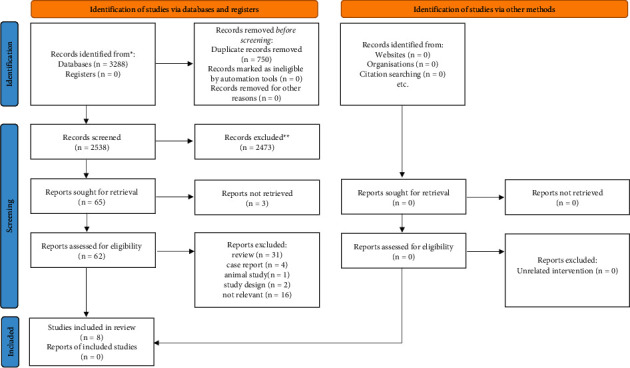
Flowchart of study selection in the systematic review.

The initial systematic literature search provided 3288 articles, where 750 records were duplicates; the remaining 2538 articles were screened. After the screening of titles and abstracts, 2473 records were excluded, and 65 articles remained for retrieval, 3 of them were not retrieved (studies with no data available after two unsuccessful requests sent to the corresponding author) and a total of 62 articles were screened through the full texts. By reading the full texts, 54 reports were eliminated as reviews (*n* = 31), animal study (*n* = 1), case report (*n* = 4), study design (*n* = 2), and not relevant (*n* = 16). A total of 8 studies met the inclusion and exclusion criteria and were preferred for data extraction (Figure 2). Our systematic review included 6 RCTs and 2 retrospective cohort studies consisted of 950 participants diagnosed by DIC. Two studies used International Society on Thrombosis and Hemostasis (ISTH) diagnostic criteria for DIC diagnosis in which platelets, PT, fibrinogen, and D-dimer were included [13, 14]. Four other articles used the Japanese Ministry of Health and Welfare (JMHW) diagnostic criteria of DIC, including platelets, fibrinogen, FDP, and PT [9, 18–20]. Mant et al. and Gobel et al. used different diagnostic criteria of DIC with similar tests, such as prothrombin time (PT), platelet count, and fibrin/fibrinogen degradation products (FDP) [15, 21].

There was a wide range of ages from newborn to elderly between participants from 4 different countries.  Table 2 displays the details of 8 articles included in this systematic review.

### 3.2. Cohort Studies

In a study, Ning et al. have investigated the effect of heparin and LMWH on patients with coronavirus because of the risk of DIC in these patients. A total of 449 patients with severe COVID-19 were entered into the study, from which 97 patients met the ISTH criteria. Patients had been treated for 7 days, where 94 patients were treated by LMWH (40–60 mg enoxaparin/d) and 5 patients received UFH (10000–15000 U/d); also they did not receive any anticoagulants other than heparin during these 7 days or longer. Finally, they have reported a significant reduction in the 28-day mortality rate in patients treated with heparin and LMWH in comparison with nontreating with heparin group (40.0% and 64.2%, resp., *p*=0.029) [14]. In another study, 47 patients were identified as severe DIC by following criteria: Hypofibrinogenemia in the absence of a known cause other than DIC in addition to an abnormality in at least two of the following tests: platelet count, activated partial thromboplastin time (APTT), fibrin/fibrinogen degradation products (FDP) and prothrombin time (PT), and also having one or more clinical conditions predisposing the patient to DIC. In this study, heparin showed 83% mortality rate in comparison with 86% mortality rate in non-heparin-treated patients, which resulted in no significant difference between groups [15].

### 3.3. Clinical Trial Studies

**Figure 3:**
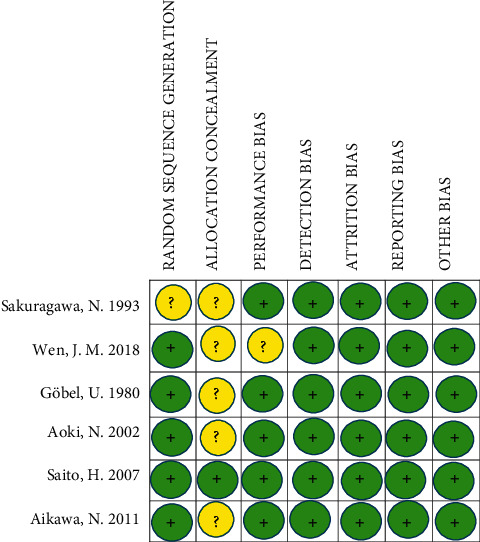
Risk of bias summary. Judgments about each risk of bias item for each included study. Circles with embedded plus sign reflect a judgment of low risk of bias. Circles with embedded question mark reflect a judgment of unclear risk of bias.

Sakuragawa et al. conducted a multicooperative double-blind trial to evaluate the clinical efficacy of LMWH on DIC in comparison with heparin. Patients have been diagnosed as having DIC by JMHW criteria treating with LMWH (*n* = 61) as an intervention group and heparin (*n* = 63) as a control group for 5 days. The mortality rate in the heparin group was 7.8% versus 0 in LMWH group. Based on the outcome of the clinical trial, LMWH had higher efficacy in the improvement of hemorrhage and organic symptoms but had no significant difference in the overall outcome [9]. Moreover, Wen et al. investigated the effect of heparin and LMWH separately against control group on traumatic DIC. Patients were diagnosed by the ISTH criteria and divided into three groups, treated by heparin (*n* = 25), LMWH (*n* = 26), and “coagulation factors only” as a control group (*n* = 26). Results showed a significant difference in mortality rate in the control group in comparison with the two treatment groups and no substantial difference was observed between heparin and LMWH groups (57.7% control, 19.2% LMWH, and 24% heparin) [13]. In another clinical trial, the effect of heparin was analyzed in the treatment of newborn infants with respiratory distress syndrome and DIC. Forty newborns with respiratory distress syndrome and DIC were enrolled in this clinical controlled double-blind study, treating with heparin or placebo. Unlike mortality rate, a major difference was observed in the duration of artificial ventilation [21]. In the other three RCTs, heparin was used as a control group and in one of these studies had a higher mortality rate in comparison with the intervention group [18–20]. Aoki et al. established a comparative double-blind randomized trial to explore the effect of activated protein C and unfractionated heparin (UHF) on DIC. They enrolled 104 patients who were diagnosed as DIC by JMHW criteria and treated with activated protein C (*n* = 49) as an intervention and heparin (*n* = 55) as a control group during 6 days. The bleeding got worsen in the 8 patients treated with heparin but not in APC receiving patients. There was no severe life-threatening bleeding in either group. Also, there was no significant difference in DIC-related organ symptoms between both groups. Ultimately, they observed a significantly lower 28-day mortality rate in the APC group in comparison with the heparin-treated group (20.4% and 40%, resp.). There were no severe adverse effects in either group [18]. In another research, Saito et al. investigated the effect of recombinant human soluble thrombomodulin (ART-123) and heparin on DIC. They screened 241 patients and randomized 234 DIC patients diagnosed by JMHW criteria during five years. In the end, the primary efficacy endpoint was assessed in 224 (ART-123: *n* = 112, heparin: *n* = 112). Finally, it was reported there is no substantial difference between ART-123 and heparin groups in the 28-day mortality rate (17.2% and 18%, resp.) [19]. As well, Aikawa et al. studied the effect of thrombomodulin alfa (TM-ALFA) and heparin on infection-induced DIC through a retrospective randomized controlled trial. They enrolled 227 patients and analyzed them by JMHW criteria. 147 patients were excluded, and the remaining 80 patients served as subjects (TM-ALFA: *n* = 42, heparin *n* = 38). Eventually, the 28-day mortality rate was lower in TM-ALFA-treated patients compared to the heparin group (21.4% and 31.6%, resp., representing an absolute difference of 10.2% (95% CI of difference, 9.1% to 29.4%)). Also, the DIC resolution rate was assessed using both JMHW and Japanese Association for Acute Medicine (JAAM) criteria. The DIC resolution rate assessed by JMHW criteria for TM-ALFA and heparin was reported as 73.2% and 63.2% (95% CI, −10.5% to 30.5%) and by JAAM criteria as 67.5% and 55.6% (95% CI, −9.8% to 33.7%), respectively [20]. The quality of RCTs was assessed by Cochrane Collaboration's tool, and the result is reported in Figure 3. There were two studies that we excluded as they did not consider a control group [22, 23]. Summary and the result of 8 included studies have been shown in Table 2.

### 3.4. Therapeutic Effect of Heparin and Its Preparations in Pre-DIC

In addition, we identified four articles that included pre-DIC patients in their studies [24–27]. Pawlowski et al. examined the effect of heparin and LMWH through a retrospective cohort study on COVID-19 patients. Since coagulopathies are a main category among the complications of COVID-19 especially in a critical care setting, a wide range of anticoagulants, such as heparin and LMWH, are being used in this field. The 28-day mortality rate significantly decreased in patients treated with LMWH (3.7%) compared with the heparin group (17%) (*p* value < 0.05). The rate of DIC incidence was lower in LMWH group in comparison with heparin group (0% and 1%, resp.). So, they have reported that LMWH is more efficient in reducing the 28-day mortality rate and incidence of DIC in COVID-19 patients [27]. In another prospective cohort study, Cheng et al. analyzed the effects of LMWH and UFH on patients with exertional heat stroke (EHS) with thrombocytopenia. They evaluated the 28-day mortality rate and incidence of DIC in 64 patients with EHS. Severe EHS may cause many complications including DIC, acute respiratory distress syndrome, and multiple organ dysfunction syndrome (MODS) [28, 29]. In this study, patients were treated by LMWH and UFH for 5 days possessed lower but not significant difference in the 28-day mortality rate in the LMWH group compared with UFH-treated group (24.2% and 32.3%, resp., *p*=0.78). Also, there was not a substantial difference in the incidence of DIC between the two groups (LMWH: 15.2%, UFH: 12.9%, *p*=1) [26]. Also, Liu et al. evaluated the effect of low-dose heparin as a treatment for early DIC during sepsis through a prospective clinical study. Patients treated with heparin needed a shorter duration of artificial ventilation and fewer days in the ICU in comparison with the control group (*p*=0.048 and 0.017, resp.). Also, it has been observed that the patients treated with heparin showed significant lesser incidence of DIC (control: 40%, heparin: 9.1%; *p* value = 0.034) but no significant difference in the 28-day mortality rate (control: 40%, heparin: 31.8%; *p* value = 0.434) [25]. In another prospective cohort study, conducted by El-Nawawy et al., the effect of early diagnosis and management of pre-DIC on high-risk group of patients at PICU was evaluated. For definite DIC treatment, the positive D-dimer subgroup received four different regimes of remedy: no specific therapy (*n* = 9); plasma substitution only (*n* = 9); plasma substitution + heparin therapy (*n* = 9); and plasma substitution + heparin + tranexamic acid (*n* = 9). The most reduction in 28-day mortality rate was seen in plasma substitution + heparin + tranexamic acid group as compared with no specific therapy, plasma substitution only and plasma substitution + heparin therapy (33%, 100%, 77%, and 100%, resp., *p* value = 0.0014) [24].

## 4. Discussion

The current study identified heparin as a therapeutic option in patients with DIC, especially in pre-DIC conditions. Nonetheless, more evaluation of therapeutic approaches seems to be necessary to find a desirable treatment [30]. The impact of heparin and its preparations on DIC mortality rate was evaluated in a total of 144 patients of two cohort studies. In a retrospective cohort study with good quality of assessment, the efficacy of heparin and LMWH has been investigated in patients with coronavirus and showed a significant high mortality rate in nontreating heparin group as compared with heparin group [14]. In another cohort study with poor-quality assessment, heparin presented 83% mortality rate, in comparison with 86% mortality rate in non-heparin-treated patients, indicating no significant difference between the two groups [15]. In this study, 34 patients had critical failure of one or more organ systems in addition to DIC and the effective role of heparin can be considered in earlier stages of DIC. It should be mentioned that this cohort had a smaller population and lower quality NOS score in comparison with the first cohort study. In two RCT studies, the effect of LMWH was investigated alone or with heparin in separate groups. Interestingly, both of them demonstrated that heparin and its preparations have a significant effect on the reduction of mortality rate in DIC patients [9, 13]. Sakuragawa et al. reported a 7.8% mortality rate in heparin group and no mortality was observed in the LMWH group. However, there was no significant difference between groups [9]. Another clinical trial stated a significant difference in mortality rate between control group and LMWH-heparin group [13]. According to these two RCTs, heparin and its preparations can reduce mortality rate in DIC patients due to their exceptional mechanisms through inhibition of thrombin and reducing coagulation disorder. In another RCT, heparin did not reduce the mortality rate in comparison with the placebo group but significantly decreased the duration of artificial ventilation in postpartum shock or respiratory distress in newborns [21]. Although heparin did not decrease the mortality rate, a shorter length of artificial ventilation requirement was provided in survived newborns with respiratory distress syndrome and DIC. Three recent RCTs have investigated the effects of various components against heparin as a control group. Aoki et al. explored the effect of activated protein C and UHF on DIC, and they reported a lower 28-day mortality rate in the APC group [18]. Another study examined the effect of ART-123 and heparin on DIC and they observed no significant difference between ART-123 and heparin groups in the 28-day mortality rate [19]. Also, Aikawa et al. stated higher but not significant mortality rate with heparin in comparison with that of thrombomodulin alfa [20]. In these three RCTs, heparin showed no higher significant mortality rate and harmful effect in comparison with other interventional agents except one.

Regarding our findings, there have been some studies that evaluated the effect of heparin and its preparations on the earlier stage of DIC. The early manifestation of DIC is not well known. The hypercoagulable condition of early DIC has recently been termed pre-DIC and diagnosed by a predisposing factor for DIC and fibrinolytic coagulation defects. Early diagnosis and management of pre-DIC condition may avoid the incidence of DIC and decrease the mortality rate [25]. We found four articles that included pre-DIC patients in their researches [24–27]. As shown in the four recent studies, early administration of heparin in patients with DIC can be more effective to reduce the mortality rate and hospitalization. Heparin mediated its anticoagulant effect by its engagement with ATIII which makes a conformational change in ATIII and so strikingly accelerates its capability to inactivate the thrombin (factor IIa), factor Xa, and factor IXa. The most susceptible coagulation enzyme to heparin-ATIII complex activity is thrombin. Heparin also inhibits the thrombin through another plasma cofactor, heparin cofactor II (HCII) with no requirement for ATIII binding [31]. As thrombin is a key component in the DIC pathogenesis, it seems to be a rational approach to managing DIC with heparin and its preparative. In addition to the extreme hypercoagulability state, inflammation plays a substantial role in DIC pathogenesis [8]. Recent studies observed a convincing anti-inflammatory effect from heparin as a result of its capability to downregulate and inhibit the activity of many cytokines such as IFN*γ* and IL-6, especially in earlier stages of inflammatory conditions [32]. Thus, heparin can be used in DIC management with antithrombin and anti-inflammatory properties. To the best of our knowledge, our study is the first systematic review of the RCTs and retrospective cohort studies due to any underlying disease that determines the effect of heparin and its preparations on DIC mortality and duration of hospitalization. Several limitations should be stated for the present study. The first limitation of our study was the small number of studies that have investigated the effect of heparin and its preparations on DIC mortality and hospitalization. The second limitation was the high heterogeneity of the study populations and the intervention/control groups. Due to this high heterogeneity of the studies and lack of valuable effect size data, we were not able to perform a meta-analysis. More clinical trials are needed to investigate the effect of heparin on earlier stages of DIC.

## 5. Conclusions

Altogether, it can be concluded that administration of heparin and its preparations in DIC patients could reduce the mortality rate and duration of hospitalization, especially if its administration could be started in earlier stages.

## Figures and Tables

**Table 1:** Clinical conditions associated with DIC.

Clinical conditions triggering DIC	Causes of DIC
Sepsis or severe infection	Potentially any microorganism but particularly gram-negative bacteria
	Viral infections (i.e., viral hemorrhagic fever)
	Malaria
	Rickettsia infection

Malignancy	Hematological malignancies (acute promyelocytic leukemia)
	Solid tumors (pancreatic, stomach, colorectal cancer, and mucin-secreting adenocarcinoma)

Trauma	Head trauma
	Severe tissue injury
	Burns
	Fat embolism
	Surgery
	Heat stroke of shock

Vascular abnormalities	Giant hemangiomas (Kasabach–Merritt syndrome)
	Aortic aneurysm
	Vasculitis

Organ destruction	Pancreatitis, severe inflammation, tissue necrosis

Obstetrical calamities	HELLP syndrome
	Amniotic fluid embolism
	Eclampsia
	Placenta previa
	Placental abruption

Liver disease	Cirrhosis
	Acute hepatic necrosis

Severe toxic or immunological reactions	Severe transfusion reactions (incompatible blood transfusion reactions)
	Snake bites (such as from those belonging to the genus *Echis*)
	Transplant reaction
	Graft-versus-host disease

**Table 2:** Characteristics of studies included in systematic review.

Year, author	Study design	Underlying disease	Study population	Intervention	Mortality rate (%)	Control	Mortality rate (%)	*p* value	Diagnostic criteria	Quality of study	Result
Tang et al., 2020	Retrospective cohort study	COVID-19-sepsis	97	LMWH-heparin	40	No heparin exposure	64.2	0.029	ISTH	Good	Effective
Mant and King, 1979	Retrospective cohort study	Infections, shock, trauma, hepatic disease, malignancy	47	Heparin	83	No heparin exposure	86	>0.05	OC	Poor	Neutral
Sakuragawa et al., 1993	Randomized controlled clinical trial	Malignant tumor, Infection, Vascular disease, gynecologic disease, surgical disease, burn	124	LMWH (Dalteparin)	0	Heparin	7.8	NS	JMHW	FIGURE 2	Effective
Wen et al., 2018	Randomized controlled clinical trial	Trauma	77	LMWH/heparin	19.2/24	Coagulation factors only	57.7	<0.05/<0.05	ISTH	FIGURE 2	Effective
Göbel et al., 1980	Randomized controlled clinical trial	Postpartum shock or respiratory distress	40	Heparin	31.5	Placebo	29.4	NR	OC	FIGURE 2	Neutral
Aoki et al., 2002	Randomized controlled clinical trial	APL, cancer, infection	104	Activated protein C	20.4	Heparin	40	<0.05	JMHW	FIGURE 2	NotEffective
Saito et al., 2007	Randomized controlled clinical trial	Malignancy or infection	224	Recombinant human soluble thrombomodulin	17.2	Heparin	18	>0.05	JMHW	FIGURE 2	Neutral
Aikawa et al., 2011	Retrospective RCT	Infection	80	Thrombomodulin alfa	21.4	Heparin	31.6	NR	JMHW	FIGURE 2	Neutral

## Data Availability

The data that support the findings of this study are available from the corresponding author upon reasonable request.

## Supplementary Materials

Supplementary Materials The search strategy has been uploaded.
