# Supplementary material for: The Effect of Heparin and Its Preparations on Disseminated Intravascular Coagulation Mortality and Hospitalization: A Systematic Review
Source: Int J Clin Pract. 2022 Jul 9;2022:2226761. doi: 10.1155/2022/2226761 (PMC9288321; doi:10.1155/2022/2226761)
Supplement: Supplementary Materials — The search strategy has been uploaded. [file 2226761.f1.docx]

PubMed

(“Disseminated intravascular coagulation” [Mesh] OR “disseminated intravascular clotting”[TIAB] OR “disseminated intravascular coagulation” [TIAB] OR “intravascular disseminated coagulation” [TIAB] OR “consumption coagulopathy” [TIAB] OR “coagulopathy consumption” [TIAB] OR “consumption coagulopathies” [TIAB] OR “coagulation disseminated intravascular” [TIAB] OR “disseminated intravascular coagulations” [TIAB]) AND ("Heparin"[Mesh] OR “Unfractionated Heparin” [TIAB] OR “Heparin, Unfractionated” [TIAB] OR “Sodium Heparin” [TIAB] OR “Heparin, Sodium” [TIAB] OR “Heparin Sodium” [TIAB] OR “alpha-Heparin” [TIAB] OR “alpha Heparin” [TIAB] OR "UH"[TIAB] OR "UFH"[TIAB] OR "Heparin, Low-Molecular-Weight"[Mesh] OR "Low-Molecular-Weight Heparin"[TIAB] OR "LMWH" [TIAB] OR “Low Molecular Weight Heparin” [TIAB] OR “Enoxaparine” [TIAB] OR “Dalteparin” [TIAB] OR “Tinzaparin” [TIAB]) AND (Hospitalization[Mesh] OR hospitaliz*[TIAB] OR “Patient Admission”[Mesh] OR “patient admission*”[TIAB] OR “Patient Readmission”[Mesh] OR “patient readmission*”[TIAB] OR Mortality[Mesh] OR mortality*[TIAB] OR “mortality rate*”[TIAB] OR “death rate*”[TIAB])

Results: 158

EMBASE

('disseminated intravascular clotting':exp OR 'disseminated intravascular clotting':ab,ti OR ‘blood coagulation’:ab,ti OR ‘disseminated intravascular; clotting’:ab,ti OR ‘intravascular; coagulopathy, consumption’:ab,ti OR ‘consumption coagulopathy’:ab,ti OR ‘consumptive coagulopathy’:ab,ti OR ‘consumptive thrombohaemorrhagic disorder’:ab,ti OR ‘consumptive thrombohemorrhagic disorder’:ab,ti OR ‘defibrination syndrome; defibrination’:ab,ti OR ‘DIC syndrome’:ab,ti OR ‘diffuse intravasal coagulation’:ab,ti OR ‘diffuse intravascular blood coagulation’:ab,ti OR ‘diffuse intravascular clotting’:ab,ti OR ‘diffuse intravascular coagulation’:ab,ti OR ‘diffuse intravascular coagulopathy’:ab,ti OR ‘disseminated fibrin thromboembolism’:ab,ti OR ‘disseminated intravasal blood coagulation’:ab,ti OR ‘disseminated intravasal clotting’:ab,ti OR ‘disseminated intravasal coagulation’:ab,ti OR ‘disseminated intravasal coagulopathia’:ab,ti OR ‘disseminated intravasal coagulopathy’:ab,ti OR ‘disseminated intravasal thromboembolism’:ab,ti OR ‘disseminated intravascular blood clotting’:ab,ti OR ‘disseminated intravascular blood coagulation’:ab,ti OR ‘disseminated intravascular coagulation’:ab,ti OR ‘disseminated intravascular coagulopathy’:ab,ti OR ‘disseminated intravascular thromboembolism’:ab,ti OR ‘fibrin thromboembolism, disseminated’:ab,ti OR ‘intravasal agglutination’:ab,ti OR ‘intravasal blood clotting’:ab,ti OR ‘intravasal clotting’:ab,ti OR ‘intravascular blood clotting’:ab,ti OR ‘intravascular clotting’:ab,ti OR ‘intravascular coagulation’:ab,ti OR ‘intravascular coagulopathy’:ab,ti OR ‘intravascular diffuse coagulation’:ab,ti OR ‘intravascular disseminated blood coagulation’:ab,ti OR ‘intravascular disseminated clotting’:ab,ti OR ‘intravascular disseminated coagulation’:ab,ti OR ‘intravascular disseminated coagulopathy’:ab,ti OR ‘intravenous coagulation’:ab,ti OR ‘massive intravasal coagulopathy’:ab,ti) AND ('heparin':exp OR 'heparin':ab,ti OR 'low molecular weight heparin':exp OR 'low molecular weight heparin':ab,ti OR ‘Unfractionated Heparin’:ab,ti OR ‘Heparin, Unfractionated’:ab,ti OR ‘Sodium Heparin’:ab,ti OR ‘Heparin, Sodium’:ab,ti OR ‘Heparin Sodium’:ab,ti OR ‘alpha-Heparin’:ab,ti OR ‘alpha Heparin’:ab,ti OR ‘UH’:ab,ti OR ‘UFH’:ab,ti OR ‘LMWH’:ab,ti OR ‘Enoxaparine’:ab,ti OR ‘Dalteparin’:ab,ti OR ‘Tinzaparin’:ab,ti) AND (‘Hospitaliz*’ OR ‘Patient Admission*’ OR ‘Patient Readmission*’ OR Mortality* OR ‘mortality rate*’ OR ‘death rate*’)

Results: 1116

SCOPUS

( TITLE-ABS-KEY ( *"disseminated intravascular clotting"* )  OR  TITLE-ABS-KEY ( *"disseminated intravascular clotting"* )  OR  TITLE-ABS-KEY ( *"blood coagulation"* )  OR  TITLE-ABS-KEY ( *"disseminated intravascular; clotting"* )  OR  TITLE-ABS-KEY ( *"intravascular; coagulopathy, consumption"* )  OR  TITLE-ABS-KEY ( *"consumption coagulopathy"* )  OR  TITLE-ABS-KEY ( *"consumptive coagulopathy"* )  OR  TITLE-ABS-KEY ( *"consumptive thrombohaemorrhagic disorder"* )  OR  TITLE-ABS-KEY ( *"consumptive thrombohemorrhagic disorder"* )  OR  TITLE-ABS-KEY ( *"defibrination syndrome;defibrination"* )  OR  TITLE-ABS-KEY ( *"DIC syndrome"* )  OR  TITLE-ABS-KEY ( *"diffuse intravasal coagulation"* )  OR  TITLE-ABS-KEY ( *"diffuse intravascular blood coagulation"* )  OR  TITLE-ABS-KEY ( *"diffuse intravascular clotting"* )  OR  TITLE-ABS-KEY ( *"diffuse intravascular coagulation"* )  OR  TITLE-ABS-KEY ( *"diffuse intravascular coagulopathy"* )  OR  TITLE-ABS-KEY ( *"disseminated fibrin thromboembolism"* )  OR  TITLE-ABS-KEY ( *"disseminated intravasal blood coagulation"* )  OR  TITLE-ABS-KEY ( *"disseminated intravasal clotting"* )  OR  TITLE-ABS-KEY ( *"disseminated intravasal coagulation"* )  OR  TITLE-ABS-KEY ( *"disseminated intravasal coagulopathia"* )  OR  TITLE-ABS-KEY ( *"disseminated intravasal coagulopathy"* )  OR  TITLE-ABS-KEY ( *"disseminated intravasal thromboembolism"* )  OR  TITLE-ABS-KEY ( *"disseminated intravascular blood clotting"* )  OR  TITLE-ABS-KEY ( *"disseminated intravascular blood coagulation"* )  OR  TITLE-ABS-KEY ( *"disseminated intravascular coagulation"* )  OR  TITLE-ABS-KEY ( *"disseminated intravascular coagulopathy"* )  OR  TITLE-ABS-KEY ( *"disseminated intravascular thromboembolism"* )  OR  TITLE-ABS-KEY ( *"fibrin thromboembolism, disseminated"* )  OR  TITLE-ABS-KEY ( *"intravasal agglutination"* )  OR  TITLE-ABS-KEY ( *"intravasal blood clotting"* )  OR  TITLE-ABS-KEY ( *"intravasal clotting"* )  OR  TITLE-ABS-KEY ( *"intravascular blood clotting"* )  OR  TITLE-ABS-KEY ( *"intravascular clotting"* )  OR  TITLE-ABS-KEY ( *"intravascular coagulation"* )  OR  TITLE-ABS-KEY ( *"intravascular coagulopathy"* )  OR  TITLE-ABS-KEY ( *"intravascular diffuse coagulation"* )  OR  TITLE-ABS-KEY ( *"intravascular disseminated blood coagulation"* )  OR  TITLE-ABS-KEY ( *"intravascular disseminated clotting"* )  OR  TITLE-ABS-KEY ( *"intravascular disseminated coagulation"* )  OR  TITLE-ABS-KEY ( *"intravascular disseminated coagulopathy"* )  OR  TITLE-ABS-KEY ( *"intravenous coagulation"* )  OR  TITLE-ABS-KEY ( *"massive intravasal coagulopathy"* ) )  AND  ( TITLE-ABS-KEY ( *"heparin"* )  OR  TITLE-ABS-KEY ( *"low molecular weight heparin"* )  OR  TITLE-ABS-KEY ( *"Unfractionated Heparin"* )  OR  TITLE-ABS-KEY ( *"Heparin, Unfractionated"* )  OR  TITLE-ABS-KEY ( *"Sodium Heparin"* )  OR  TITLE-ABS-KEY ( *"Heparin, Sodium"* )  OR  TITLE-ABS-KEY ( *"Heparin Sodium"* )  OR  TITLE-ABS-KEY ( *"alpha-Heparin"* )  OR  TITLE-ABS-KEY ( *"alpha Heparin"* )  OR  TITLE-ABS-KEY ( *"UH"* )  OR  TITLE-ABS-KEY ( *"UFH"* )  OR  TITLE-ABS-KEY ( *"LMWH"* )  OR  TITLE-ABS-KEY ( *"Enoxaparine"* )  OR  TITLE-ABS-KEY ( *"Dalteparin"* )  OR  TITLE-ABS-KEY ( *"Tinzaparin"* ) )  AND  ( TITLE-ABS-KEY ( *"Hospitaliz*"* )  OR  TITLE-ABS-KEY ( *"Patient Admission*"* )  OR  TITLE-ABS-KEY ( *"Patient Readmission*"* )  OR  TITLE-ABS-KEY ( *"Mortality*"* )  OR  TITLE-ABS-KEY ( *"mortality rate*"* )  OR  TITLE-ABS-KEY ( *"death rate*"* ) )

Results: 1670

WOS

(TS=(“*disseminated intravascular clotting*”) OR TS=(“*disseminated intravascular clotting*”) OR TS=(“*blood coagulation*”) OR TS=(“*disseminated intravascular; clotting*”) OR TS=(“*intravascular; coagulopathy, consumption*”) OR TS=(“*consumption coagulopathy*”) OR TS=("*consumptive coagulopathy*") OR TS=("*consumptive thrombohaemorrhagic disorder*") OR TS=("*defibrination syndrome;defibrination*") OR TS=("*DIC syndrome*") OR TS=("*diffuse intravasal coagulation*") OR TS=("*diffuse intravascular blood coagulation*") OR TS=("*diffuse intravascular clotting*") OR TS=("*diffuse intravascular coagulation*") OR TS=("*diffuse intravascular coagulation*") OR TS=("*diffuse intravascular coagulopathy*") OR TS=("*disseminated fibrin thromboembolism*") OR TS=("*disseminated intravasal blood coagulation*") OR TS=(“*disseminated intravasal clotting*”) OR TS=(“*disseminated intravasal coagulation*”) OR TS=("*disseminated intravasal coagulopathia*") OR TS=("*disseminated intravasal thromboembolism*") OR TS=("*disseminated intravascular blood clotting*") OR TS=("*disseminated intravascular coagulation*") OR TS=("*disseminated intravascular thromboembolism*") OR TS=("*fibrin thromboembolism, disseminated*") OR TS=("*intravasal agglutination*") OR TS=("*intravasal blood clotting*") OR TS=("*intravasal clotting*") OR TS=("*intravascular blood clotting*") OR TS=("*intravascular clotting*") OR TS=("*intravascular coagulation*") OR TS=(“*intravascular coagulopathy*”) OR TS=(“*intravascular diffuse coagulation*”) OR TS=(“*intravascular disseminated blood coagulation*”) OR TS=("*intravascular disseminated clotting*") OR TS=("*intravascular disseminated coagulation*") OR TS=("*intravascular disseminated coagulopathy*") OR TS=("*intravenous coagulation*") OR TS=("*massive intravasal coagulopathy*")) AND (TS=(“*heparin”*) OR TS=(“*low molecular weight heparin*”) OR TS=(“*Unfractionated Heparin*”) OR TS=(*“Heparin, Unfractionated”*) OR TS=(“*Sodium Heparin*”) OR TS=(“*Heparin, Sodium*”) OR TS=(“*alpha-Heparin*”) OR TS=(“*alpha Heparin*”) OR TS=("*UH*") OR TS=("*UFH*") OR TS=("*LMWH*") OR TS=("*Enoxaparine*") OR TS=("*Dalteparin*") OR TS=(“*Tinzaparin*”)) AND (TS=(“*Hospitaliz**”) OR TS=("*Patient Admission**") OR TS=(“*Patient Readmission**”) OR TS=("*Mortality**") OR TS=("*mortality rate**") OR TS=("*death rate**"))

Results: 344
